# [^*18*^F]FDG PET/CT Studies in Transgenic Hualpha-Syn (A53T) Parkinson’s Disease Mouse Model of α-Synucleinopathy

**DOI:** 10.3389/fnins.2021.676257

**Published:** 2021-06-15

**Authors:** Rommani Mondal, Anthony-David Tawatao Campoy, Christopher Liang, Jogeshwar Mukherjee

**Affiliations:** Preclinical Imaging, Department of Radiological Sciences, University of California, Irvine, Irvine, CA, United States

**Keywords:** Parkinson’s disease, Hualpha-Syn A53T mice, α-synuclein, [^18^F]FDG PET/CT, hypometabolism, brain, spinal cord, limb muscle

## Abstract

Transgenic mice line M83 that express the A53T mutant α–synuclein protein at six times the level of endogenous mice α–synuclein are a model of α-synucleinopathy found in Parkinson’s disease (PD). This Hualpha-Syn (A53T) PD model is useful in assessing non-motor deficits at earlier stages of onset of PD. We report findings on metabolic changes using [^18^F]FDG PET/CT in the Hualpha-Syn (A53T) PD mouse model in comparison to non-carrier mice. Whole-body PET/CT imaging of male and female mice were carried out 2 h after [^18^F]FDG ip administration under 3% isoflurane anesthesia. Brain images were analyzed with PET images coregistered to a mouse brain MRI template. Hualpha-Syn (A53T) mice had significantly lower [^18^F]FDG uptake in several brain regions compared to the no-carrier mice. Significant hind limb muscle and lower spinal cord [^18^F]FDG hypometabolism at 9 months of age in A53T PD mice was also indicative of neurodegenerative disease, with a progressive motoric dysfunction leading to death. Significant decrease (up to 30%) in [^18^F]FDG uptake were observed in 9-month old male and female Hualpha-Syn (A53) mice. This is consistent with the cortical hypometabolism in PD patients. Hualpha-Syn (A53) mice may thus be a suitable model for studies related to PD α-synucleinopathy for the discovery of new biomarkers.

## Introduction

Parkinson’s disease (PD) is a neurodegenerative disease which manifests in motor and non-motor ailments leading to cognitive impairment. The sporadic nature of PD development can make studying specific pathologies challenging (Gasser, 2009). The endogenous protein α-synuclein, is generally structured as a random coil but in PD, the misfolded α-synuclein usually aggregates in neurons (Levigoureux et al., 2019). Aggregation of misfolded α-synuclein in intracellular inclusions, Lewy bodies (LB), are some of the hallmarks of human PD (Martí et al., 2003).

Transgenic mice models of α-synucleinopathy are now available to help understand progression of PD (Martin et al., 2006). The Hualpha-Syn (A53T) transgenic mice exhibit the familial PD associated A53T missense mutant form of human α-synuclein and express the A53T mutant α-synuclein at a level sixfold that of the endogenous mice α-synuclein (Giasson et al., 2002). The hemizygous mice spontaneously develop neurodegenerative disease between 9 and 16 months of age (Paumier et al., 2013). Neuronal abnormalities displayed by affected mice include pathological accumulations of α-synuclein and ubiquitin. In the A35T α-Syn mutant mice, it was discovered that they attain intraneuronal inclusions, mitochondrial degeneration, and cell death in the neocortex, brainstem, and spinal cord. In addition, they formed inclusions similar to LB in neurons, and had profound deficits in their motor neurons, which could explain the paralysis in A53T line G2-3 mice (Martin et al., 2006). The A53T mice also develop fine, sensorimotor, and synaptic deficits before developing age-related gross motor and cognitive impairment (Paumier et al., 2013). Rat models of PD have been developed by expression of A53T α-synuclein in the substantia nigra causing nigrostriatal degeneration (Koprich et al., 2010).

Glucose metabolism studies using [^18^F]FDG-PET have been successfully carried out in human PD (Ma et al., 2007; Matthews et al., 2018). With [^18^F]FDG-PET, specific patterns of deficit in the PD brain can be identified (Buchert et al., 2019). The precision in the topographic and functional analysis of [^18^F]FDG-PET imaging of brain metabolism confirms its utility for understanding PD (Trošt et al., 2019). Thus, [^18^F]FDG-PET has been used in small animal models of PD in an attempt to understand alterations in brain metabolism in disease progression. Rat models of α-synuclein rAAV2/7 PD have been studied using [^18^F]FDG-PET imaging (Devrome et al., 2019). In combination with other PET radiotracers, [^18^F]FDG-PET has been studied in mice models (Levigoureux et al., 2019). Since transgenic A53T mice is a good model of α-synucleinopathy and is useful in assessing non-motor deficits at earlier stages of onset of PD, we have carried out this comparative [^18^F]FDG-PET/CT study of transgenic A53T and non-carrier male and female mice.

## Materials and Methods

### Animals

The Hualpha-Syn (A53T) transgenic line M83 mice strain [Tg(Prnp-SNCA^∗^A53T)83Vle/J; stock no. 004479; 4 male and 4 female] and non-carrier mice [4 male and 4 female; Tg(Prnp-SNCA^∗^A53T)23Mkle/J/#006823] were purchased from Jackson Laboratory. All mice were born on 01/21/2020. At 7 to 9 months of age, weight of female A53T mice were 20–26 *g*, non-carrier female was 20–28 *g* and male A53T mice weighed 26–34 *g* and non-carrier male were 30–38 *g*. All mice were housed in sterilized cages. Non-carrier animals did not exhibit any abnormal motor activity whereas one male and two female A53T mice had hind limb paralysis and were euthanized (Supplementary Table 1). All animals recovered from the anesthesia required for the PET/CT imaging procedures. Animal studies were approved by the Institutional Animal Health Care and Use Committee of University of California, Irvine.

### Equipment

An Inveon dedicated PET scanner (Siemens Medical Solutions, Knoxville, TN, United States), which has a resolution of 1.46 mm in the center of the field-of-view, was used for the PET studies (Constantinescu and Mukherjee, 2009). An Inveon Multimodality (MM) CT scanner (Siemens Medical Solutions, Knoxville, TN, United States) was used for CT acquisitions and attenuation correction in the combined PET/CT experiments. A Sigma Delta anesthetic vaporizer (DRE, Louisville, KY, United States) was used to induce and maintain anesthesia during intraperitoneal injections of [^18^F]FDG and PET/CT acquisitions.

### Experimental Protocol

Male (*n* = 4) and female (*n* = 4), hemizygous Hualpha-Syn (A53T) and no-carrier male (*n* = 4) and female (*n* = 4) mice were used in the study. Mice were imaged twice, at 7 and 9 months. All mice were fasted for at least 24 h prior to the imaging study. All mice were injected [^18^F]FDG (PETNET solutions) intraperitoneally in normal saline (7.4 ± 0.7 MBq in 0.05–0.1 mL sterile saline) under 3% isoflurane (St. Joseph, MO, United States; Coleman et al., 2017). Mice were then awake after [^18^F]FDG injections and free to move in their cages for 2 h. They were placed in the supine position in a mouse holder and anesthetized with 3% isoflurane for whole-body PET/CT imaging. A 15 min-long PET scans was acquired 2 h after [^18^F]FDG injections followed by a 10-min-long CT scan after the PET scan for attenuation correction and anatomical delineation of PET images. The Inveon Multimodality scanner was used for all acquisitions in combined PET/CT experiments.

### PET/CT Imaging

**FIGURE 1:**
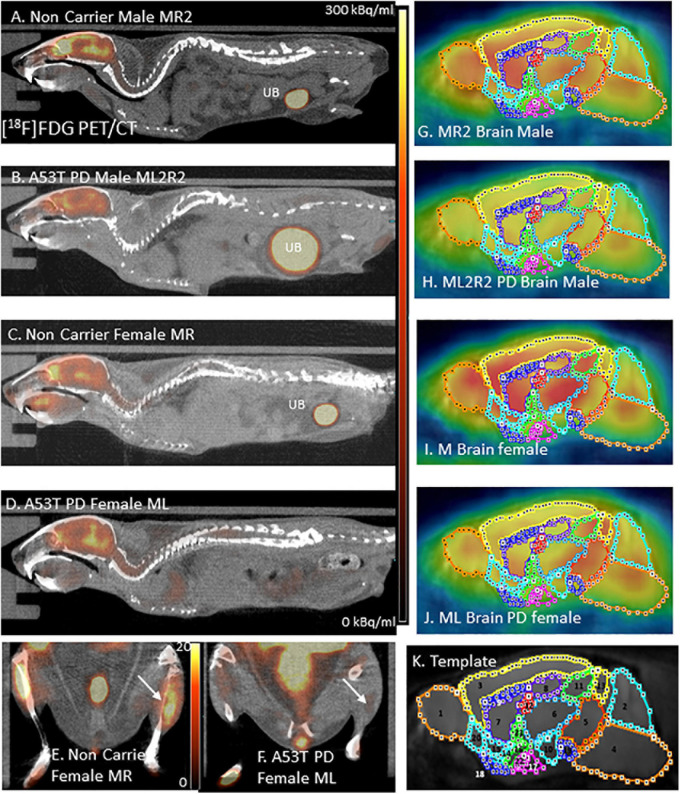
**(A)** [^18^F]FDG PET/CT full body mouse image of Control Male MR2; UB, Urinary bladder. **(B)** [^18^F]FDG PET/CT full body mouse image of PD Male ML2R2; **(C)** [^18^F]FDG PET/CT full body mouse image of Control Female MR; **(D)** [^18^F]FDG PET/CT full body mouse image PD Female ML; **(E)** Hind limb [^18^F]FDG PET/CT of non-carrier female MR mouse; **(F)** Hind limb [^18^F]FDG PET/CT of PD female ML mouse; **(G–J)** [^18^F]FDG images coregistered with MR template regions; and **(K)** Mouse MR template showing regions: 1. Olfactory bulb; 2. Cerebellum; 3. Neocortex; 4. Brainstem; 5. Midbrain; 6. Thalamus; 7. Caudate-Putamen; 8. Hippocampus; 9. Corpus Collusum; 10. Hypothalamus; 11. Superior colliculi; 12. Ventricles; 13. Inferior colliculi; 14. Ventral pallidum; 15. Nucleus accumbens; 16. Substantia innominate; 17. Amygdala; 18. Olfactory tubercle; 19. Substantia nigra; 20. Piriform area; 21. Globus pallidus; and 22. Magnocellular nucleus.

The Inveon PET and MM CT scanners were placed in the “docked mode” for combined PET/CT experiments. PET data were reconstructed as 128 × 128 × 159 matrices with a transaxial pixel of 0.776 mm and slice thickness of 0.796 mm using an OSEM3D algorithm (2 OSEM iterations, 18 MAP iterations, and 1.5 target resolution). PET images were corrected for random coincidences, attenuation and scatter. All images were calibrated in units of Bq/cm 3 by scanning a Ge-68 cylinder (6 cm diameter) with known activity and reconstructing the acquired image with parameters identical to those of [^18^F]FDG images. The CT images were spatially transformed to match the reconstructed PET images (Figures 1A–F). The CT projections were acquired with the detector-source assembly rotating over 360 degrees and 720 rotation steps. A projection bin factor of 2 was used in order to increase the signal to noise ratio in the images. The CT images were reconstructed using cone-beam reconstruction with a Shepp filter with cutoff at Nyquist frequency and a binning factor of 2 resulting in an image matrix of 512 × 512 × 1,008 and a voxel size of 0.052 mm.

### Image Analysis

All *in vivo* images were analyzed using Inveon Research Workplace (IRW) software (Siemens Medical Solutions, Knoxville, TN, United States) and PMOD Software (PMOD Technologies, Zurich, Switzerland). Whole-body PET/CT images were analyzed using the IRW software for [^18^F]FDG uptake and any other CT anomalies in the whole body images. For brain quantitative analysis, brain images were analyzed using PMOD, with PET images coregistered to a mouse brain MRI template (Figure 1K). Using the template, volumes-of-interest (VOI) were drawn on PET images (Figures 1G–J) using previously described methods (Coleman et al., 2017). The magnitude of [^18^F]FDG was expressed as standard uptake value (SUV) which was computed as the average[^18^F]FDG activity in each volume of interest, VOI (in kBq/mL) divided by the injected dose (in MBq) times the body weight of each animal (in Kg). Male and female animals were analyzed as two separate groups and were further divided into control and PD groups for a total of 4 groups. The SUV values in the male and female was then statistically analyzed using students *t*-test and A53T PD mice were compared with non-carrier mice. A *p* value of <0.05 was considered to indicate statistical significance.

For muscle analysis on the CT scans, VOIs were drawn as irregular contours on the high resolution CT images of the A53T mice and compared to the non-carrier mice. Hounsfield units (HU) were measured of the hind limb muscles using the IRW software.

## Results

Hind limb paralysis in three A53T mice (1 male, 2 females) was observed between 8 and 11 months. The mice were not able to access food and water and were euthanized. At 9 months there was a significant decrease (>50%) in hind limb muscle [^18^F]FDG in the A53T mice (Figure 1F) compared to the non-carrier mice (Figure 1E). The remaining A53T mice at 12 months did not show issues with movement, but whole body CT scans of the mice revealed weakening of the hind limb muscles compared to the non-carrier mice. The CT scans revealed approximately 40% HU reduction in female A53T mice compared to non-carrier mice, at 12 months of age, suggesting significant muscle loss (Supplementary Table 2). Limb paralysis in the A53T mice has been previously reported (Giasson et al., 2002).

**FIGURE 2:**
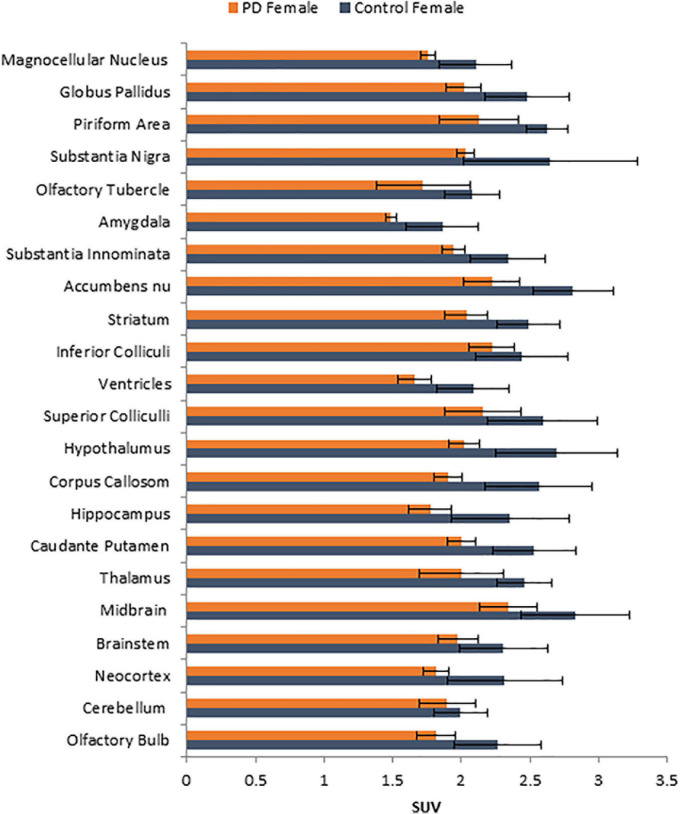
Comparison of average [^18^F]FDG standard uptake values (SUV) with standard deviation of female non-carrier mice with A53T PD female mice across different brain regions shown in Figure 1K.

**FIGURE 3:**
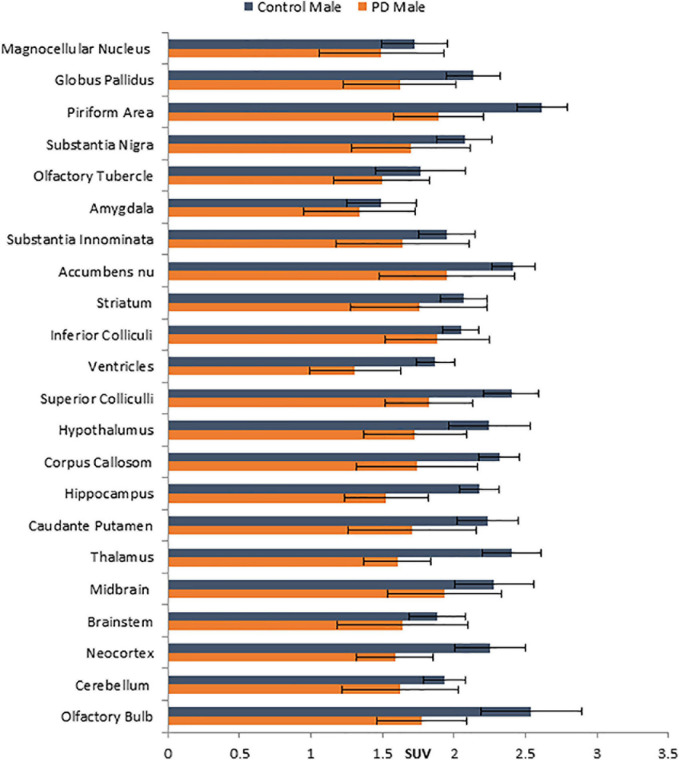
Comparison of average [^18^F]FDG standard uptake values (SUV) with standard deviation of male non-carrier mice with A53T PD male mice across different brain regions shown in Figure 1K.

No unusual uptake of [^18^F]FDG was observed in any peripheral organs in any of the A53T and non-carrier mice except for the hind limbs and lower spinal cord. Excretion of [^18^F]FDG largely occurred into the urinary bladder as expected (Figures 1A–D). Brain exhibited high levels of [^18^F]FDG uptake as can be seen in the whole body PET/CT scans (Figures 1A–D). Detailed brain analyses of [^18^F]FDG (Figures 1G–J) showed Hualpha-Syn (A53) mice had lower [^18^F]FDG uptake in all brain regions compared to the non-carrier mice at 9 months (Table 1). Female mice had more brain regions with significant differences compared to male mice (Figures 2, 3). Additionally, in this limited study, female exhibited greater hypometabolism in the hind limbs as well as muscle weakness compared to the male mice. The olfactory bulb, neocortex, thalamus, caudate putamen, hippocampus, corpus callosum, hypothalamus, substantia nigra were some of the regions which had close to or greater than 20% reduction in [^18^F]FDG in the A53T mice compared to the non-carrier mice (Table 1 and Supplementary Figure 1).

**TABLE 1:** [^18^F]FDG brain region SUV and percent change in A53T PD mice^a^.

Brain region	Female control	Female PD	Female% difference^b^	Male control	Male PD	Male% difference^b^
Olfactory bulb	2.27	1.82	−19.86**	2.54	1.78	−30.00*
Cerebellum	2.00	1.90	−4.97	1.94	1.62	−16.30
Neocortex	2.32	1.81	−21.71**	2.25	1.59	−29.40*
Brainstem	2.31	1.98	−14.46*	1.88	1.64	−12.90
Midbrain	2.83	2.34	−17.17*	2.28	1.93	−15.21
Thalamus	2.46	2.00	−18.68*	2.41	1.60	−33.28*
Caudate putamen	2.53	2.00	−21.10**	2.24	1.71	−23.52
Hippocampus	2.36	1.77	−24.79**	2.18	1.53	−29.78*
Corpus callosum	2.56	1.90	−25.80**	2.32	1.74	−24.82
Hypothalamus	2.69	2.02	−24.87**	2.25	1.73	−23.06
Superior colliculi	2.59	2.16	−16.65*	2.40	1.82	−23.98*
Inferior colliculi	2.44	2.22	−8.88*	2.05	1.88	−8.00
Ventral pallidum	2.49	2.04	−18.18**	2.07	1.76	−15.05
Accumbens nucleus	2.82	2.22	−21.08**	2.42	1.95	−19.29
Substantia innominata	2.34	1.95	−16.78**	1.95	1.64	−16.04
Amygdala	1.86	1.49	−19.96**	1.49	1.34	−10.44
Olfactory tubercle	2.08	1.72	−17.07	1.76	1.50	−15.14
Substantia nigra	2.65	2.03	−23.40***	2.07	1.70	−18.05
Piriform area	2.62	2.13	−18.83*	2.62	1.89	−27.66*
Globus pallidus	2.48	2.02	−18.57**	2.13	1.62	−24.00
Magnocellular nucleus	2.11	1.76	−16.51***	1.72	1.49	−13.27

*^*a*^Results are from 9-month old mice.*

^*b*^*P*-value: * < 0.05; ** < 0.005; and *** < 0.0005.

The hemizygous mice develop adult-onset neurodegenerative disease between 9 and 16 months of age, with a progressive motoric dysfunction leading to death. The [^18^F]FDG PET/CT scans at 7 months showed some decreases, but at 9 months these changes were significant reductions. These brain regions (cortex, hippocampus, brain stem, and others) were shown to accumulate α-synuclein aggregates after 6 months (Paumier et al., 2013). Immunostaining of brain sections of A53T PD mice and non-carrier mice brains using Millipore anti- α-synuclein polyclonal antibody (#AB5038) were carried out in the Ventana benchmark ultra (Trimmer et al., 2004). Supplementary Figure 2B shows accumulation of α-synuclein aggregates in the cortex of A53T PD mice compared to non-carrier mice (Supplementary Figure 2A).

## Discussion

Rodent models of motor deficits of PD, either by 1-methyl-4-phenyl-1,2,3,6-tetrahydropyridine (MPTP) treatment in mice or 6-hydroxy-DOPA in rats, causing loss of dopaminergic neurons in the striatum have been used in PET studies. We previously reported use of [^18^F]fallypride (a dopamine D2/D3 postsynaptic receptor PET radiotracer) in an MPTP-treated mouse model to investigate effects of exercise (Vuckovic et al., 2010). Development of α-synucleinopathy models allows studies related to the non-motor deficits observed in PD (Martí et al., 2003; Paumier et al., 2013). Progressive accumulation of α-synuclein aggregates has been shown in the cortex, midbrain, brain stem and other brain regions in the A53T model and may be reflective of the α-synucleinopathy in human PD. This accumulation of α-synuclein aggregates in the A53T mice manifests itself in cognitive and behavior deficits (Giasson et al., 2002). Thus it appears that the A53T mice would be a good PD model to study human PD and for use in biomarker development. Although dopaminergic PET imaging has been used extensively in PD patients, [^18^F]FDG PET allows measurement of cortical deficits in PD (Walker et al., 2018).

Human PD patients exhibit hypometabolism in several brain regions including the cortex, and [^18^F]FDG PET has been considered a useful imaging tool for idiopathic PD (Walker et al., 2018). Our results show a significant brain reduction of [^18^F]FDG uptake at 9 months of age. Both male and female mice exhibit hypometabolism, changes in the female mice were significant in more brain regions. Neocortex, hippocampus, thalamus and olfactory bulb were some regions in both males and females that were significantly reduced. One preliminary study showed [^18^F]FDG hypometabolism in the A53T mice line M83 after intrathecal administration of α-synuclein preformed fibrils (DeDuck et al., 2020).

Our previous studies have shown [^18^F]FDG uptake in mice muscles (Mirbolooki et al., 2014) similar to our findings in the non-carrier mice in this study. The A53T PD mice muscles, however, have a significant reduction in [^18^F]FDG uptake and weakening of the hind limb muscles was evident in the CT scans of the A53T mice progressing to hind limb paralysis. These findings suggest that A53T mice may be a suitable model for PET radiotracer development to study progression of muscle weakening and limb paralysis. Inclusion-body myositis (IBM) may be caused by α-synuclein (Askanas et al., 2000). It remains to be confirmed if the A53T PD mice muscle weakness may be associated with IBM. Our initial evaluation of the spinal cord in the A53T PD mice suggests lower levels of [^18^F]FDG uptake in the lumbosacral regions compared to the non-carrier mice (Supplementary Figure 3). However, more detailed analysis is required to evaluate the metabolic consequences of α-synucleinopathy in the spinal cord known to contain dopamine receptors (Kaur et al., 2014).

Our additional studies in the A53T PD mice model will include studies of the serotonergic 5-HT1A receptors since these receptors may be affected in the hippocampus of A53T PD mice (Deusser et al., 2015). We propose to use [^18^F]mefway for PET imaging of the serotonin 5-HT1A receptor (Mukherjee et al., 2016). Nicotinic α4β2 receptors play a role in cognitive impairment and may be targets for neuroprotection in PD (Perez-Lloret and Barrantes, 2016). Our goal is to use [^18^F]nifene (Mukherjee et al., 2018) and [^18^F]nifrolene (Pichika et al., 2013) to understand changes in nicotinic acetylcholinergic mechanisms in the A53T PD mice. Our previous work on dopamine D2/D3 receptors in the MPTP mice model of PD found exercise-induced increases in the receptor (Vuckovic et al., 2010). We plan to use [^18^F]fallypride (Mukherjee et al., 2015) to study potential changes in D2/D3 receptors in the A53T PD mice in order to assess changes in these receptors with respect to the hypometabolism reported here. Such studies have been reported with other mice models of PD (Levigoureux et al., 2019). The A53T PD mice will be an excellent model for evaluation of potential PET radiotracer candidates for aggregated α-synuclein and in understanding the pathophysiology of PD.

In conclusion, our study shows (1) Hypometabolism in brain regions of the A53T α-synucleinopathy PD model; (2) Hypometabolism and weakening of the muscles leading to limb paralysis; and (3) Potential hypometabolism in sacrolumbar regions of the spinal cord. A limitation of this study is the small number of animals in each group. The loss of 37% of the A53T mice due to limb paralysis within a 12-month period in this preliminary feasibility study provides insights on planning a larger study to evaluate additional biomarkers using PET/CT.

## Data Availability Statement

The original contributions presented in the study are included in the article/Supplementary Material; further inquiries can be directed to the corresponding author/s.

## Ethics Statement

The animal study was reviewed and approved by University of California, Irvine.

## Author Contributions

JM: study concept and design, obtained funding, and study supervision. CL and JM: acquisition of data. A-DC, CL, RM, and JM: analysis and interpretation of data and drafting of the manuscript. A-DC: statistical analysis. All authors had full access to all the data in the study and take responsibility for the integrity of the data and the accuracy of the data analysis.

## Conflict of Interest

The authors declare that the research was conducted in the absence of any commercial or financial relationships that could be construed as a potential conflict of interest.

## Supplementary Material

Supplementary Figure 1 Comparison of difference between non-carrier and A53T PD mice in SUV units in males and females of [^18^F]FDG uptake in different brain regions.

Supplementary Figure 2 Immunostained cortex brain regions in non-carrier mice **(A)** and A53T PD mice **(B)** using Millipore anti-α-synuclein polyclonal antibody (#AB5038). Greater accumulation of α-synuclein aggregates were observed in the A53T PD mice.

Supplementary Figure 3 Hypometabolism measured by [^18^F]FDG uptake in the lumbosacral regions (arrows) of the A53T PD mice **(B)** compared to the non-carrier mice **(A)**.

Supplementary Table 1 Details of non-carrier mice and A53T PD mice used in the study.

Supplementary Table 2 CT analysis of non-carrier mice and A53T PD mice.
