# Supplementary figures and images for: [18F]FDG PET/CT Studies in Transgenic Hualpha-Syn (A53T) Parkinson’s Disease Mouse Model of α-Synucleinopathy
Source: Front Neurosci. 2021 Jun 15;15:676257. doi: 10.3389/fnins.2021.676257 (PMC8239288; doi:10.3389/fnins.2021.676257)

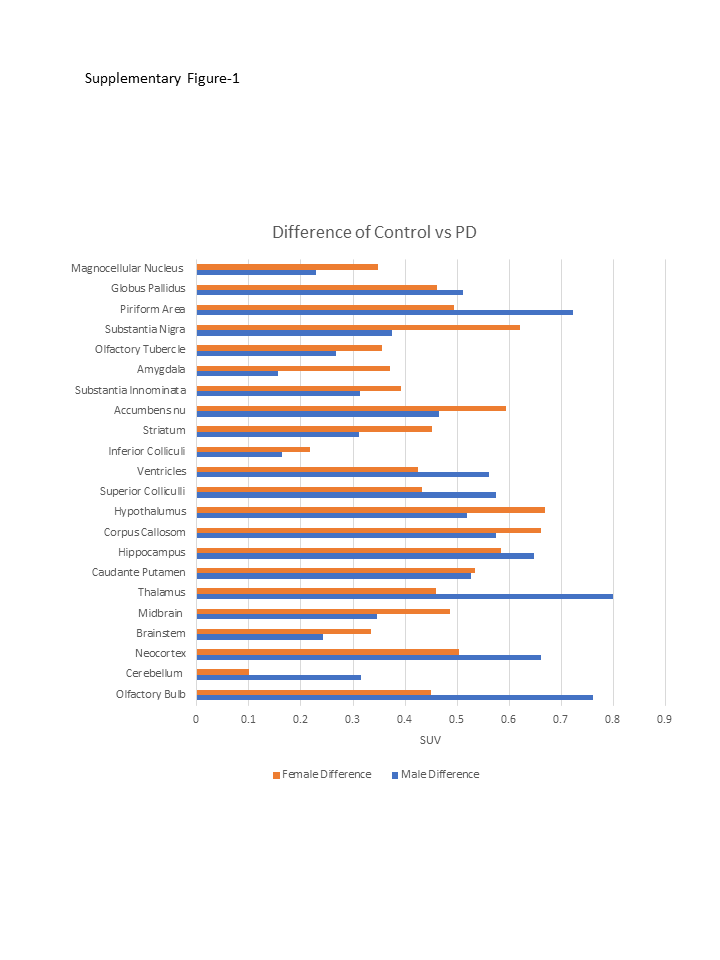

Supplement: Supplementary Figure 1 — Comparison of difference between non-carrier and A53T PD mice in SUV units in males and females of [18F]FDG uptake in different brain regions. [file Image_1.TIF]

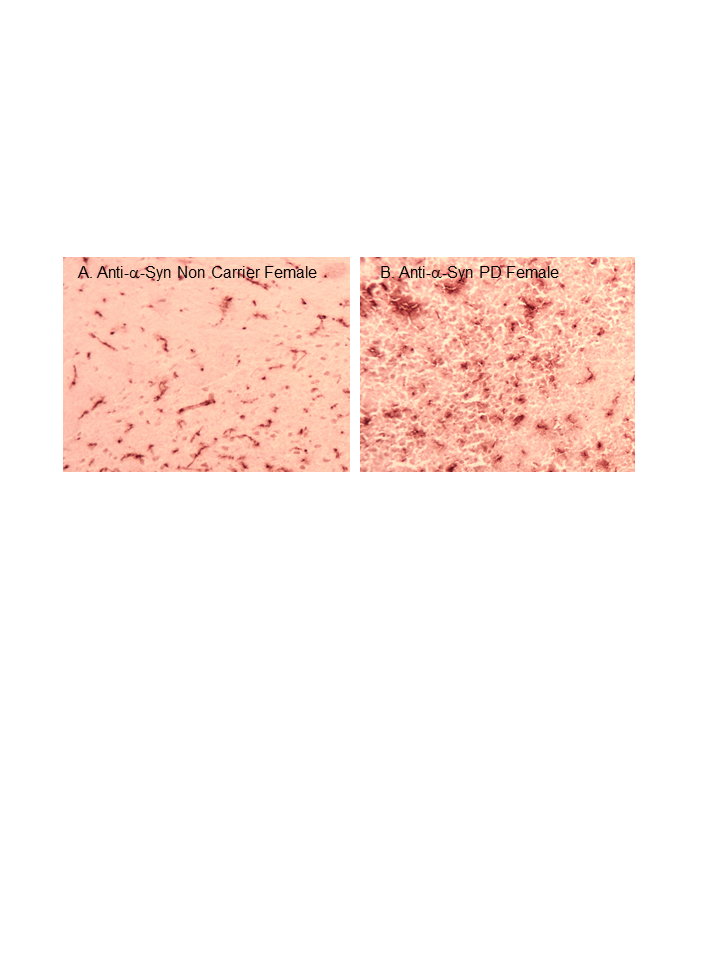

Supplement: Supplementary Figure 2 — Immunostained cortex brain regions in non-carrier mice (A) and A53T PD mice (B) using Millipore anti-α-synuclein polyclonal antibody (#AB5038). Greater accumulation of α-synuclein aggregates were observed in the A53T PD mice. [file Image_2.TIF]

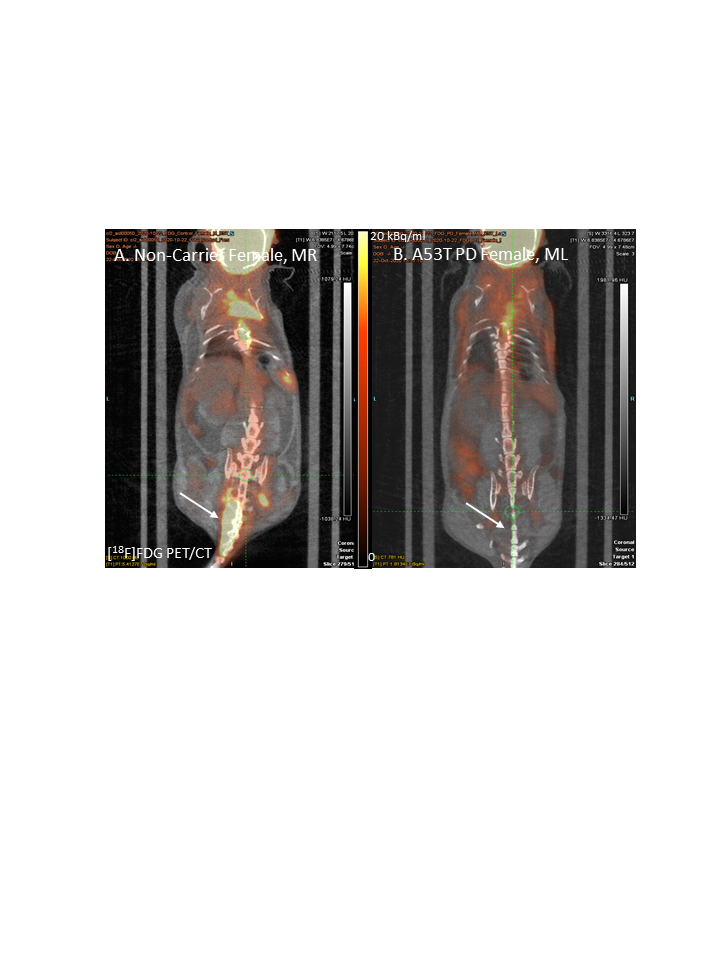

Supplement: Supplementary Figure 3 — Hypometabolism measured by [18F]FDG uptake in the lumbosacral regions (arrows) of the A53T PD mice (B) compared to the non-carrier mice (A). [file Image_3.TIF]
