# Supplementary material for: [18F]FDG PET/CT Studies in Transgenic Hualpha-Syn (A53T) Parkinson’s Disease Mouse Model of α-Synucleinopathy
Source: Front Neurosci. 2021 Jun 15;15:676257. doi: 10.3389/fnins.2021.676257 (PMC8239288; doi:10.3389/fnins.2021.676257)
Supplement: Supplementary Table 1 — Details of non-carrier mice and A53T PD mice used in the study. [file Table_1.DOCX]

**Supplementary Table-1:** HuAlpha A53T Mice Used in the Study

| **Serial No** | **A53T/NC**  **Gender^1^** | **DOB^2^**  **Ear tag** | **Weight^3^** | **PET/CT Study^4^ and**  **Mice Observations** |
| --- | --- | --- | --- | --- |
| 1 | NC; F | M | 22-26g | Normal |
| 2 | NC; F | MR | 22-28g | Normal |
| 3 | NC; F | MR2 | 24-26g | Normal^5^ |
| 4 | NC; F | ML1 | 20-22g | Normal |
| 5 | NC; M | MR3 | 32-38g | Normal |
| 6 | NC; M | MR2 | 30-36g | Normal |
| 7 | NC; M | ML2R1 | 36-42g | Normal |
| 8 | NC; M | ML2 | 34-36g | Normal |
| 9 | A53T; F | MLR | 22-26g | Hind Limb paralysis^6^ |
| 10 | A53T; F | ML2 | 20-22g | Hind Limb paralysis^7^ |
| 11 | A53T; F | ML1 | 22-24g | Impaired balance^8^ |
| 12 | A53T; F | MR | 20-22g | Impaired balance^8^ |
| 13 | A53T; M | ML1R2 | 26g | Hind Limb paralysis^9^ |
| 14 | A53T; M | M | 26-28g | Normal |
| 15 | A53T; M | MR | 26-28g | Normal |
| 16 | A53T; M | ML2R2 | 32-34g | Normal |

^1^Transgenic A53T and non-carrier (NC) mice were obtained from Jackson’s Lab.

^2^All animals were born on 01/21/20.

^3^Weight ranges of mice are over a 4 month period.

^4^[^18^F]FDG 6845±740 kBq in 0.05-0.1 mL injected intraperitoneally.

^5^Mouse euthanized on 1/14/21 after two [^18^F]FDG PET/CT scans

^6^Mouse euthanized on 12/17/20 after two [^18^F]FDG PET/CT scans

^7^Mouse euthanized on 1/4/21 after two [^18^F]FDG PET/CT scans

^8^Slow, sluggish movement in cage, otherwise normal.

^9^Mouse euthanized on 9/15/20 after one [^18^F]FDG PET/CT scans
