# Supplementary material for: [18F]FDG PET/CT Studies in Transgenic Hualpha-Syn (A53T) Parkinson’s Disease Mouse Model of α-Synucleinopathy
Source: Front Neurosci. 2021 Jun 15;15:676257. doi: 10.3389/fnins.2021.676257 (PMC8239288; doi:10.3389/fnins.2021.676257)
Supplement: Supplementary Table 2 — CT analysis of non-carrier mice and A53T PD mice. [file Table_2.DOCX]

**Supplementary Table-2:** CT analysis of lower limb in HuAlpha A53T Mice

|  | **Mice** | **Lower limb,**  **HU*** | **Percentage Change** |
| --- | --- | --- | --- |
| 1 | Non-Carrier Female | 57.4 | 39.4% decrease in line M83 A53T female mice |
| 2 | Line M83 A53T Female | 34.8 |  |
| 3 | Non-Carrier  Male | 56.3 | 9.74% decrease in line M83 A53T female mice |
| 4 | Line M83  A53T Male | 50.8 |  |

^*^HU= Hounsfield units; values are from two mice, 12 months old in each group.
